# Transcriptomic profiles of poplar (*Populus simonii* × *P. nigra*) cuttings during adventitious root formation

**DOI:** 10.3389/fgene.2022.968544

**Published:** 2022-09-08

**Authors:** Yue Yu, Nan Meng, Song Chen, Hongjiao Zhang, Zhijie Liu, Yiran Wang, Yanan Jing, Yuting Wang, Su Chen

**Affiliations:** ^1^ State Key Laboratory of Tree Genetics and Breeding, Northeast Forestry University, Harbin, China; ^2^ College of Life Science, Northeast Forestry University, Harbin, China

**Keywords:** adventitious root, poplar, leafy cuttings, transcriptome analysis, differentially expressed genes

## Abstract

The formation of adventitious roots (ARs) is vital for the vegetative propagation of poplars. However, the relevant mechanisms remain unclear. To reveal the underlying molecular mechanism, we used RNA-seq to investigate the transcriptional alterations of poplar cuttings soaked in water for 0, 2, 4, 6, 8, and 10 d; 3,798 genes were differentially expressed at all the time points, including 2,448 upregulated and 1,350 downregulated genes. Biological processes including “cell cycle,” “photosynthesis,” “regulation of hormone levels,” and “auxin transport” were enriched in the differentially expressed genes (DEGs). KEGG results showed that the common DEGs were most enriched in the pathway of “Carbon fixation in photosynthetic organisms” and “Starch and sucrose metabolism.” We further dissected 38 DEGs related to root and auxin, including two *lateral root primordium* 1 (*LRP1*), one *root meristem growth factor* (*RGF9*), one *auxin-induced in the root* (*AIR12*), three rooting-associated genes (*AUR1* and *AUR3*), eight auxin transcription factors (*ARFs* and *LBD*s), 10 auxin respective genes (*SAUR*s and *GH3*s), nine auxin transporters (*PIN*s, *ABC*s, *LAX*2, and *AUX*s), and four auxin signal genes (*IAA*s and *TIR1*). We found that the rooting abilities of poplar cuttings with and without leaves are different. By applying different concentrations of IBA and sucrose to the top of cuttings without leaves, we found that 0.2 mg/ml IBA and 2 mg/ml sucrose had the best effect on promoting AR formation. The transcriptome results indicated photosynthesis may influence AR formation in poplar cuttings with leaves and revealed a potential regulatory mechanism of leafy cuttage from poplar cuttings. In addition, we provided a new perspective to resolve rooting difficulties in recalcitrant species.

## Introduction

Cuttage is the most efficient way for vegetative propagation of tree species because it can maintain valuable traits of woody plants (Zhao et al., 2014). However, cuttage of some tree species faces the problem of difficulty in the formation of adventitious roots (ARs) (Zhao et al., 2014; Wang Z et al., 2019). ARs play important roles during plant growth, including absorption of water and nutrients, plant stability, and stress resistance (Bellini et al., 2014; Bannoud and Bellini, 2021; Li, 2021). ARs derived from stems and leaves are affected by hormone levels and external environmental factors (Steffens and Rasmussen, 2016; De Almeida et al., 2017; Guan et al., 2019). The formation process of ARs is similar to that of lateral roots, which includes induction, initiation, and extension phases (Bellini et al., 2014). The meristematic cells neighboring cambium begin to divide into a root primordium, and gradually differentiate into the initial shape of ARs (Bellini et al., 2014). Finally, ARs penetrate the epidermal cells and develop into the root system (Bellini et al., 2014; Hilo et al., 2017). Hormone levels are the main internal factors for the induction and development of ARs (Fukaki and Tasaka, 2009), among which auxin is the most important one. In plants, auxin is mainly synthesized in leaves and transported to the bottom of plants through polar transport (Lin and Sauter, 2019; Casanova-Sáez et al., 2021). IAA (indole-3-acetic acid) mainly exists in the cytoplasm and chloroplasts of plants. In the cytoplasm, IAA undergoes synthesis and non-decarboxylation degradation (van Berkel et al., 2012). While IAA in chloroplasts is protected from the metabolism, its concentration is changed by the concentration of IAA in the cytoplasm (Damodaran and Strader, 2019; Casanova-Sáez et al., 2021). Therefore, the internal auxin concentrations in cuttings with and without leaves are different, which may have a huge impact on the induction of ARs (Dao et al., 2020). In addition, sugars are also important products of photosynthesis, which can provide energy for the development of ARs (Rapaka et al., 2005).

Because of its roles in promoting AR formation, auxin has been widely used in the cuttage of plants, especially for species with low rooting rates (An et al., 2020). IBA (indole-3-butyric acid) is the most popular auxin form used in cuttage (Frick and Strader, 2018). The transport, reception, and response of auxin signals in plants are regulated by a set of the complex molecular mechanism (Casanova-Sáez et al., 2021). The transport of auxin between plasma membranes is mediated by auxin influx and efflux carriers (Damodaran and Strader, 2019). The plant-specific PIN (Pin-formed) family protein and the ABC (ATP-binding cassette) superfamily act as efflux carriers in some herb plants (Forestan and Varotto, 2012). Auxin is perceived by two distinct classes of receptors: TIR1/AFB (transport inhibitor response 1/auxin-related F-box) and Aux/IAA (auxin/indole-3-acetic acid) co-receptors that control transcriptional responses to auxin (Shu et al., 2019; Xu et al., 2021). At the same time, Aux/IAA has a repressed effect on the expression of auxin-responsive genes such as ARFs (auxin-response factors) (Tromas et al., 2013; Zhao et al., 2014). The accumulation of auxin is beneficial to the interaction of TIR1/AFBs within SCFTIR1/AFB ubiquitin ligase E3 complexes and Aux/IAAs co-receptor, thereby promoting the ubiquitination and degradation of the latter (Dharmasiri et al., 2005; Wang and Estelle, 2014). The induction and development mechanism of ARs is similar to that of lateral roots (LRs) (Singh et al., 2020). Auxin-response factors (ARF) are transcription factors that involve auxin signaling pathways and promote the development of ARs (Liu et al., 2020; Li, 2021). In *Arabidopsis*, AFB2-IAA12/28-ARF5 and TIR1-IAA14-ARF7/19 are involved in the development of lateral roots (Bellini et al., 2014; Yue et al., 2020). ARF7/19 activates the expression of LBD16/18/29 to promote AR formation in *Arabidopsis* (Lee et al., 2019; Yue et al., 2020)*.* ARF6, ARF8, and ARF17 regulate downstream target genes *GH3.3*, *GH3.5*, and *GH3.6*, thereby controlling free IAA levels (Lee et al., 2019; An et al., 2020; Li, 2021). Related studies have shown that the development of ARs may be related to the level of IAA in plants (Xu et al., 2021). In *Populus*, the miR160a targets six PeARF17.1 and PeARF17.2 to regulate the development of ARs (Gutierrez et al., 2012; Liu et al., 2020). Some *PeARF*s showed high expression in different stages of AR development based on transcriptome results. Ranjan et al. (2022) found that *PttARF17.1* and *PttMYC2.1* negatively regulate AR formation in poplar. AUX (AUXIN RESISTANT), LAX (LIKE AUXIN RESISTANT), and SAUR (Small auxin up RNA) have also been reported to have mediated the auxin transport signal, and SAURs may be involved in cell expansion at the same time (Titapiwatanakun et al., 2009; Spartz et al., 2012; Lee et al., 2019; An et al., 2020). In *Arabidopsis*, genes related to rooting are affected by auxin, such as LRP (lateral root primordium), AIR (auxin-induced in root), and AUR (rooting-associated genes) (An et al., 2020; Singh et al., 2020). RGF (root meristem growth factor) is a key factor involved in root meristem development (Fernandez and Beeckman, 2020); *RGF1* and *RGF2* are involved in the cell division of root meristem in *Arabidopsis* (Matsuzaki et al., 2010; An et al., 2018).

*P. simonii* × *P. nigra*, also named as *P. xiaohei*, is a hybrid and commercial tree species (Ahmed et al., 2020). Because of its excellent wood quality and stress resistance, *P. xiaohei* has been widely used for afforestation in Northern China (Yu J et al., 2017; Ahmed et al., 2020). Cuttage in poplar has advantages of shorter reproduction time, and faster reproduction rate in the actual production process (Zhao et al., 2014). But cuttings of some poplar species are difficult to form ARs, especially for aspen poplars (Zhao et al., 2014; Guan et al., 2015). At present, the relevant molecular mechanisms for the development of ARs in tree species are still not clear. In the actual production process of cuttings, the methods of soaking the base of the cuttings with auxin solution and rooting powder solution are mainly used to promote rooting (Zhao et al., 2014). Auxin has been reported to regulate root access to nutrients and water (Wang H et al., 2019). Related reports claim that polar auxin transport determines AR emergence and growth in rice (Lin and Sauter, 2019). The predecessors did not report relevant cases of using polar transport of auxin from top to bottom to promote rooting of cuttings. Here we propose a hypothesis that polar transport of auxins and organic matter produced by leaves *via* photosynthesis can promote AR development. During this process, genes related to photosynthetic signals can be co-expressed with rooting and auxin signaling genes. We applied auxin from the top of cuttings to promote rooting and examine the mechanism of AR formation in poplar stem cuttings. The results presented here show that auxin positively regulates root formation in poplar cuttings and provided a new way to solve the problem of difficult rooting of poplar cuttings.

## Materials and methods

### Plant materials and growth conditions

Three-month-old plants of *P. xiaohei* grown in the greenhouse of Northeast Forestry University were cut into 12 cm in length, and each cutting contains two axillary buds and a leaf. The cuttings with different treatments including with and without leaves, different concentrations of IBA (St Louis, MO, United States), sucrose (St Louis, MO, United States), and a mixed solution of IBA and sucrose were soaked in water. After different treatments, the cuttings will continue hydroponic rooting in the greenhouse at a temperature of 26 ± 1°C and a light time of 16-h/8-h (light/dark) photoperiod. During the rooting period, different concentrations of IBA or sucrose were added to the absorbent cotton at the top of the cuttings every day, and the slow-release effect of the absorbent cotton was used to make the hormones absorbed by the cuttings. After different treatments, the rooting of the cuttings was observed at 7, 14, and 21 d, respectively.

### Different treatments of IBA, sucrose, and NPA

We used different concentrations of IBA and sucrose, and the combinations of IBA, sucrose, and NPA to investigate the effects of auxin and sucrose on promoting AR formation; 0.05, 0.1, 0.2, 0.5, and 1 mg/ml IBA (St Louis, MO, United States) and 0.5, 1, 1.5, 2, and 5 mg/ml sucrose (St Louis, MO, United States) were applied to the top of the cuttings, respectively; 0.2 mg/ml IBA + 2 mg/ml sucrose, 0.2 mg/ml IBA + 0.2 mg/ml NPA (St Louis, MO, United States), 2 mg/ml sucrose + 0.2 mg/ml NPA, and 0.2 mg/ml IBA + 2 mg/ml sucrose + 0.2 mg/ml NPA were used as different combinations; 1 ml of each of the above substances was applied to the top of the cuttings at a 24-h interval.

### Slice observation

*P. xiaohei* cuttings with different treatments at 0, 2, 4, 6, and 8 days were collected and the bases were cut into 150–300 μm transverse sections by hand. All sections were treated with the concentration of 3% phloroglucinol (Shitai Group, China) and 30% concentrated hydrochloric acid (Shitai Group, China) for 5 min, and then the rooting of the cutting base was observed by an optical microscope (Nikon lambda Group, Japan) with 40 times magnification (×40/0.9).

### Assays of hormone levels in poplar cuttings

Axillary buds and bases of cuttings with and without leaves were cut into diameter 0.5 cm segments as fresh plant materials. A fresh plant sample was harvested, immediately frozen in liquid nitrogen, and stored at −80°C until needed; 50 mg of the plant sample was weighed into a 2-ml plastic microtube and frozen in liquid nitrogen, and dissolved in 1 ml methanol/water/formic acid (15:4:1, V/V/V) (Millipore, Bradford, United States); 10 μl internal standard mixed solution (100 ng/ml) was added into the extract as internal standards (IS) for the quantification (Zhang et al., 2020). The mixture was vortexed for 10 min, then centrifugation was performed for 5 min (12,000 r/min, and 4°C), the supernatant was transferred to clean plastic microtubes, followed by evaporation to dryness and dissolved in 100 μl 80% methanol (V/V), and filtered through a 0.22-μm membrane filter for further LC-MS/MS analysis (Zhang et al., 2020). The sample extracts were analyzed using a UPLC-ESI-MS/MS system (UPLCˈExionLC™ AD https://sciex.com.cn/; MS Applied Biosystems 6500 Triple Quadrupole, https://sciex.com.cn/). The analytical conditions were as follows: LC: column, Waters ACQUITY UPLC HSS T3 C18 (100 mm × 2.1 mm i.d. 1.8 µm); solvent system, water with 0.04% acetic acid (A), acetonitrile with 0.04% acetic acid (B); gradient program, started at 5% B (0–1 min), increased to 95% B (1–8 min), 95% B (8–9 min), finally ramped back to 5% B (9.1–12 min); flow rate, 0.35 ml/min; temperature, 40°C; injection volume, 2 μl (Zhang et al., 2020).

### Assays of sucrose levels in poplar cuttings

A fresh plant sample (same as hormone measurement) was harvested, immediately frozen in liquid nitrogen, and stored at −80°C; 0.15 g of plant material and 1 ml of water were ground into powder. Both glucose standard solution and anthrone extraction reagent use a soluble sugar kit (Solarbio, China). Measurement solution was dissolved in 15 ml methanol/water/concentrated sulfuric acid (1,000:200:100) uL. A spectrophotometer was used to measure the wavelength of the solution at the wavelength of 630 and 645 nm, respectively (Georgelis et al., 2018).

### Sample preparation, cDNA library construction, and Illumina sequencing

The stem bases of cuttings with leaves (0.5–1 cm) were harvested from 0 to 10 d with a 2-day interval for RNA extraction, cDNA library construction, and RNA-sequencing. Total RNAs of the samples were extracted using the RNA pre-Pure Plant Plus Kit (TIANGEN BIOTECH, Beijing). The RNAs were assessed using Qubit Fluorometer and Agilent 2100 Bioanalyzer, and then used for cDNA library construction according to the user manual. The cDNA libraries were sequenced by the MGISEQ-2000 platform at ANOROAD-Beijing (ANOROAD, Beijing, China). We obtained 12 cDNA libraries, two replicates for each time point. After filtering out low-quality reads, all the clean sequencing data have been submitted to the NCBI SRA (Sequence Read Archive) database (Accession No. PRJNA798213).

### RNA-sequencing data analysis

Gene expression analysis was performed by using RSEM (RNA-Seq by Expectation–Maximization) pipeline. STAR (Spliced Trans Alignment to a Reference) was used to align the sequencing reads to the reference genome (*Populus trichocarpa* v4.1) with parameters recommended by RSEM (Liu et al., 2021). Gene expression levels were then determined by using the mapping results of STAR. After calculating gene expressions for all the samples, these data were combined using TMM normalization to eliminate the influence on the expression of calculated genes (Liu et al., 2021). The differentially expressed genes at different time points were determined using edge R with thresholds of FDR < 0.05 and |log_2_FC| > 1. The minimum value of count per million was set as one to filter out samples with low expression (Jiang et al., 2016; Liu et al., 2021). GO (Gene Ontology) annotations were obtained by egg NOG (evolutionary genealogy of genes: Non-supervised Orthologous Groups). GO enrichment analysis was performed by the Fisher exact test with a cutoff of FDR < 0.05 (Liu et al., 2021). Functional pathway enrichment of all common DEGs was performed using the KEGG platform (Kyoto Encyclopedia of Genes and Genomes Platform). Spearman correlation coefficient was used to build the gene co-expressed network with a correlation coefficient > 0.8 (An et al., 2020; Liu et al., 2021).

### qRT-PCR

About 1 μg of total RNA was reversed to obtain cDNA using the Primer Script™ RT reagent Kit with gDNA Eraser (RR047, Takara, Shanghai, China). Using the NCBI primer blast platform to design qRT-PCR-specific primers, the detail of the specific primer sequence is shown in Supplementary Table S8 (Liu et al., 2021). We selected PtUBQ7 as the internal control, and each time points was performed with three biological replicates. The condition of qRT-PCR was three stages (95°C 2 min one cycle, 95°C 10 s, 60°C 30 s 40 cycles, and 95°C 15 s 60°C 1 min 95°C 30 s) and process total volume of 20 μl sample by Real-Time PCR System 7800, containing 10 μl qPCR SYBR Premix ExTaq™ Mix, 0.4 μl of each primer (10 uM), 2 μl of cDNA, and 7.2 μl ddH_2_O (Liu et al., 2021).

## Results

### The process of adventitious root formation in poplar cuttings

**FIGURE 1:**
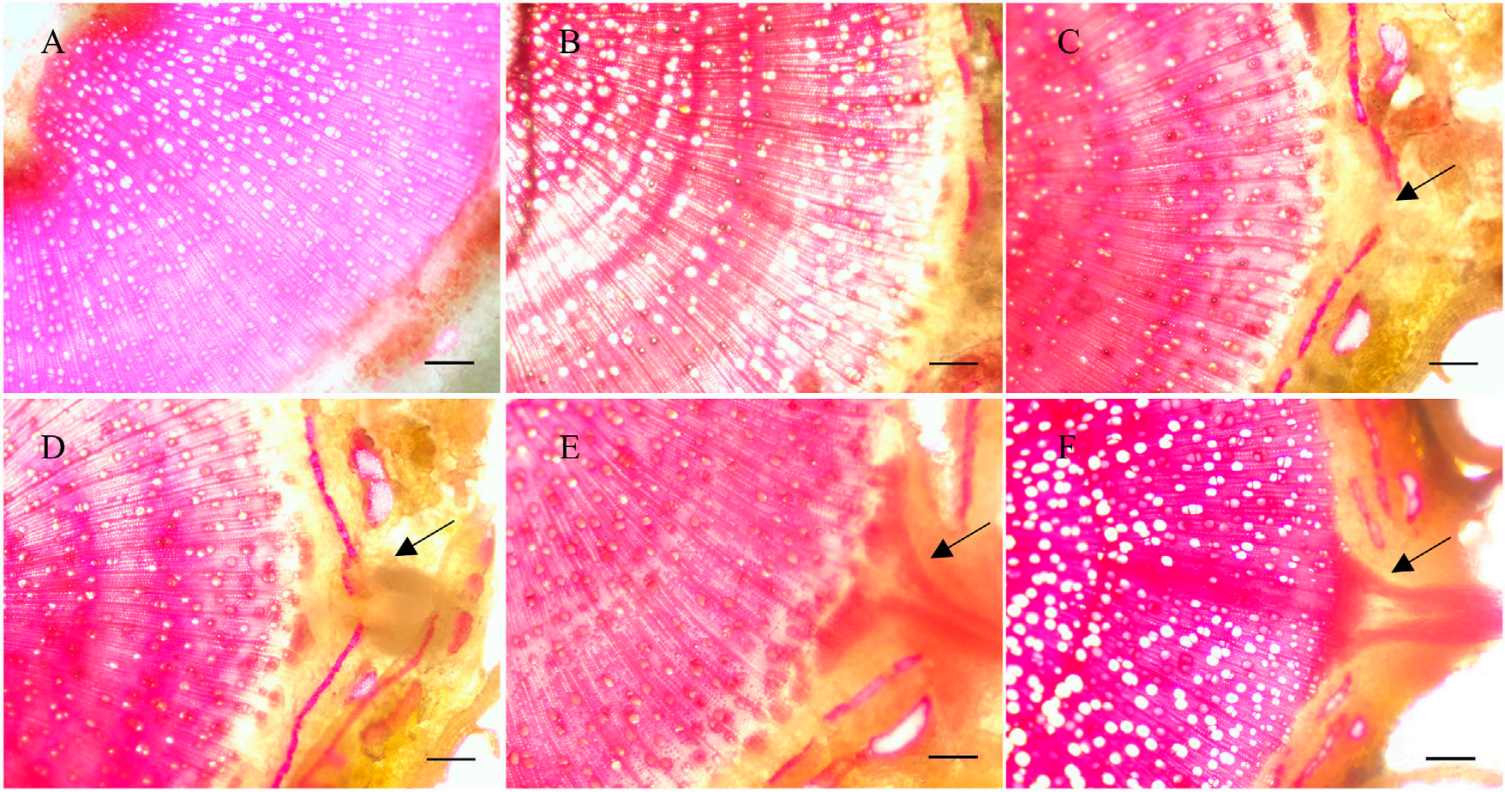
Dissection of root formation in *Populus xiaohei* cuttings treated with water. **(A–F)** Cross-sectional view of the base of *P. xiaohei* cuttings for 0, 2, 4, 6, 8, and 10 days. Arrows indicate the development area of root primordium cells between the cambium and epidermis (bars = 100 μm).

The formation of ARs in poplar cuttings is mainly related to the cell division in vascular tissues and the appearance of root primordia (Qi et al., 2019; Li et al., 2020). To obtain the original position and formation process of ARs in *P. xiaohei* cuttings, cross sections of the bases were stained with phloroglucinol and observed using an optical microscope. By cross-cutting the bases of *P. xiaohei* cuttings soaked in water, we found that the xylem, cambium, and epidermis showed a gradual and orderly layered structure within 4 days (Figures 1A,B). When the cuttings were soaked in water for 4 days (Figure 1C), the area indicated by the arrows began to swell and became a potential area for the differentiation of ARs. When the cuttings were soaked in water for 6 days (Figure 1D), the root primordium cells developed into the primary form of ARs and extended outward into the cortex. Root primordium cells continued to divide and penetrated the epidermis to form ARs (Figure 1E). Finally, the mature ARs continued to elongate and gradually improve the internal structure (Figure 1F). The induction and development process of ARs damaged the epidermal structure of the stem, which caused the outer stem structure to break off.

### Identification of differentially expressed genes during AR formation

**FIGURE 2:**
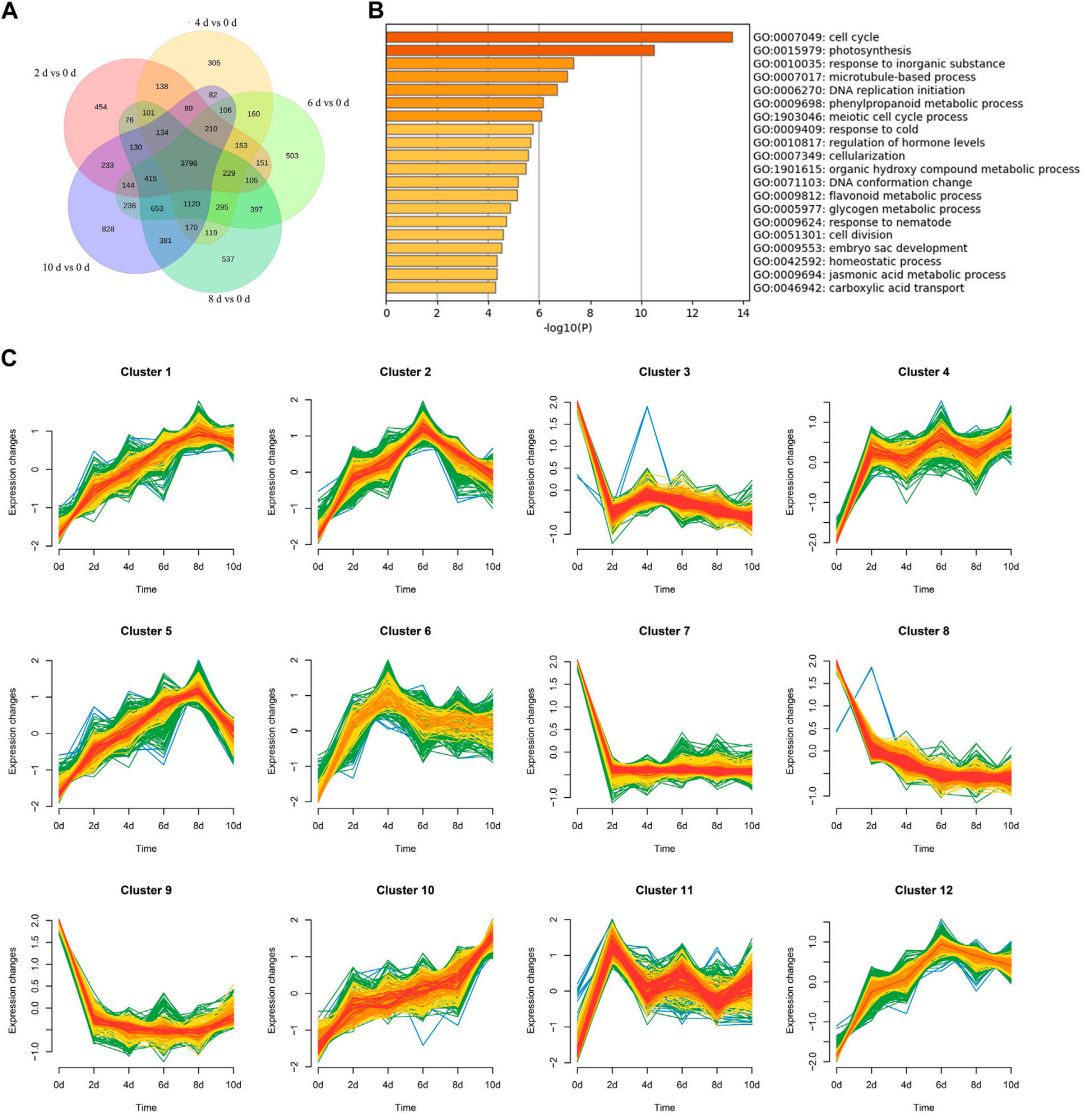
Venn maps, Go analysis, and clustering analysis of common DEGs. **(A)** 0 d as control. Number of DEGs between 0 d and different days. **(B)** Go analysis of 3,798 common DEGs. Terms with a *p*-value < 0.05, Log10(*p*) as enrichment standard. **(C)** Clustering analysis of 3,798 common DEGs.

To reveal the molecular mechanism underlying AR formation, we used RNA-seq (Liu et al., 2021) to investigate the transcriptional alterations of *P. xiaohei* cuttings soaked in water for 0, 2, 4, 6, 8, and 10 d, respectively. A total of 12 cDNA libraries, two replicates for each time point, were constructed for paired-end sequencing, and 708.9 million raw reads were generated (Table 1). After filtering out low-quality reads, a total of 705.6 million clean reads remained, accounting for an average of 98.6% in each sample (Table 1). The clean reads were aligned to the reference genome of *P. trichocarpa* by STAR (Spliced Trans Alignment to a Reference), and the average reads mapping rate was 82.91% (Table 1). Using 0 d as control, 6,560, 7,209, 8,675, 8,660, and 8,729 DEGs were identified for 2, 4, 6, 8, and 10 d, respectively. Of these DEGs, 3,798 were identified at all the time points (Figure 2A), including 2,448 upregulated and 1,350 downregulated genes (Supplementary Table S1). To better visualize the potential specific expression patterns within the DEGs, we conducted a clustering analysis of 3,798 DE genes across 18 samples according to similar expression patterns using the R package Mfuzz (Kumar and Futschik, 2007). Finally, we separated all the DEGs into 12 clusters as shown in Figure 2C. Clusters 1, 2, 4, 5, 6, 10, 11, and 12 all have an overall increasing trend of gene expression, containing 287, 375, 290, 378, 235, 286, 298, and 301 genes, respectively. Clusters 3, 7, 8, and 9 all have an overall decreasing trend, containing 372, 374, 361, and 241 genes, respectively.

**TABLE 1:** Summary of the alignment statistics.

Sample ID	Raw reads	Clean reads	Mapped reads	Mapped percent (%)
CK_rep1	29231634	29190358	24932839	85.41
CK_rep2	31208284	31138845	26602547	85.43
D2_rep1	31119844	30962150	24964378	80.63
D2_rep2	30247416	30041106	22698896	75.56
D4_rep1	30335030	30073746	25345986	84.28
D4_rep2	31681141	31472467	26451852	84.05
D6_rep1	29852391	29784325	24270441	81.49
D6_rep2	27808640	27750014	22546039	81.25
D8_rep1	29735858	29703374	25061151	84.37
D8_rep2	27366613	27307123	22705943	83.15
D10_rep1	26872805	26728099	22556779	84.39
D10_rep2	29273417	29217973	24833117	84.99

### GO and KEGG analyses

GO enrichment analysis was performed on these common DEGs to identify biological processes (BPs) involved in AR formation. The significantly enriched BPs included “cell cycle,” “photosynthesis,” and “response to an inorganic substance,” which contained 119, 52, and 140 DEGs and were enriched 2.47, 2.56, and 1.8 folds, respectively (Figure 2B). The enriched BPs also include “microtubule-based process,” “regulation of hormone levels,” “cell division,” “auxin transport,” and “jasmonic acid (JA) metabolic process” (Supplementary Table S2). Plant hormones, especially auxins, are vital factors for AR formation in plants (Li, 2021). During the AR formation in *P. xiaohei* cuttings, the BPs of “auxin transport,” “regulation of hormone levels,” and “organic acid transport” included 23, 60, and 28 DEGs, and were enriched 3.12, 2.13, and 3.34 times, respectively. It is also reported that changes in the JA level may affect the auxin content, thereby mediating the development of ARs (Gutierrez et al., 2012). Seventeen DEGs related to the “JA metabolic process” were found in the common DEGs with 4.46-fold enrichment. Some BPs that may play roles during the transition from meristem cells to root primordium were enriched. BPs of “cell division” and “microtubule-based process” included 44 and 56 DEGs, and were enriched 2.67 and 2.37 times, respectively. In addition, some biological processes related to secondary metabolites were enriched during AR formation; 23 DEGs were found in the “flavonoid metabolic process.” To further reveal the relationships between these BPs, the enriched BPs were rendered as a network plot. The BPs with a similarity > 0.3 were connected by edges (Supplementary Figure S1). The results indicated that the formation of ARs in *P. xiaohei* cuttings is complex and involved in many BPs.

**FIGURE 3:**
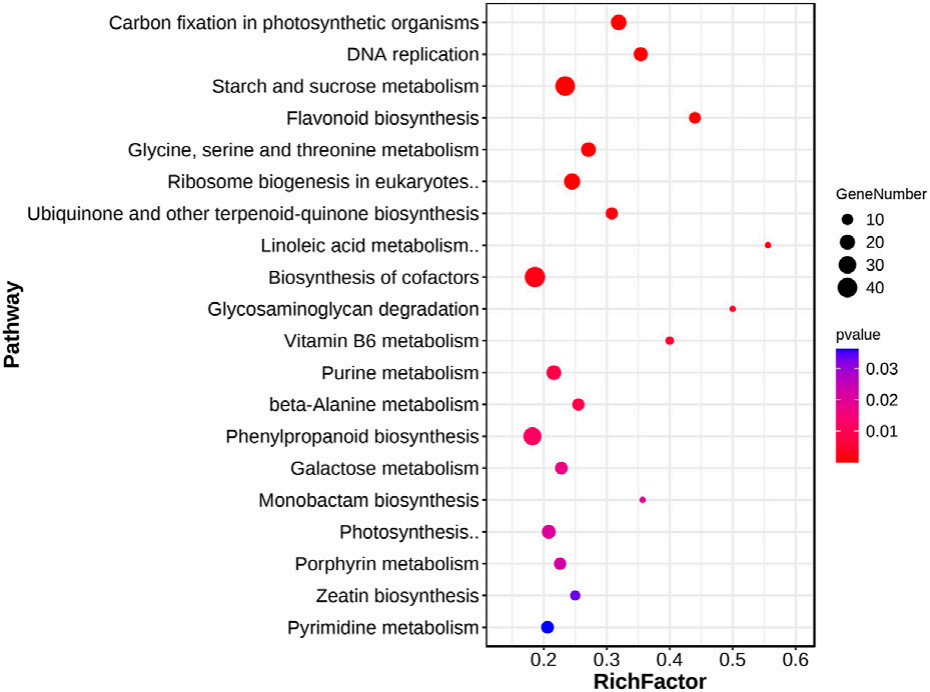
KEGG analysis of common DEGs. KEGG analysis of 3,798 common DEGs. The blot color and size represent the significance and number of DEGs enriched for this pathway, respectively. Terms with a *p*-value < 0.05.

KEGG enrichment analysis was also performed to identify enriched pathways. The results showed that the common DEGs were significantly enriched in 22 pathways (Supplementary Table S3). The significantly enriched KEGG pathways mainly related to the process of photosynthesis, including “carbon fixation in photosynthetic organisms,” “biosynthesis of cofactors,” and “starch and sucrose metabolism” (Figure 3). Starch and sucrose are important products of photosynthesis, which release energy through metabolism to promote plant organ development (Hyndman et al., 1981; Rapaka et al., 2005). In addition, we also found some other enriched pathways related to hormones and metabolites, including “flavonoid biosynthesis” and “zeatin biosynthesis.” The GO and KEGG results indicated that hormones and organic substances may play an important role in poplar cuttings during AR formation.

### Transcriptional profiles of AR formation-related genes

**FIGURE 4:**
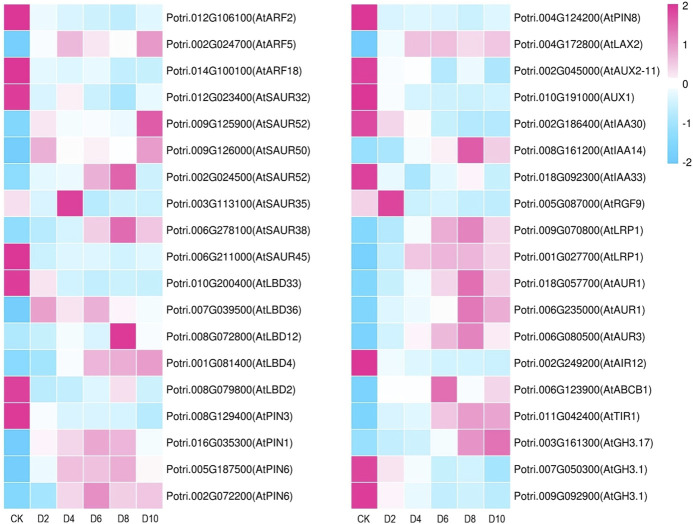
Heatmap of the expression of common DEGs related to root development and auxin signal during AR formation in poplar cuttings. D2, 4, 6, 8, 10, and CK represents 2, 4, 6, 8, 10, and 0 days of hydroponics cuttings for RNA-seq, respectively.

Since *Arabidopsis* is the most well-studied plant, we used its annotations to further dissect the common DEGs (Swarbreck et al., 2008); 260 differentially expressed TFs were first identified (Supplementary Table S4), which included MYB, ERF, ARF, AP2, LBD, NAC, and SRS families (Table 2). ARF is an important TF family which may affect the development of ARs through binding to auxin-responsive elements (Yang et al., 2019; Liu et al., 2020; Li, 2021). In this study, three differentially expressed *ARFs* were identified. The expression level of *Potri.002G024700* (best hit to *AT1G19850.1*, *ARF5*) showed a continuously raising profile and was increased around 1.8–2 times for all stages. *LBDs* are mainly regulated by ARFs and are responsible for the development of lateral organs (Lee et al., 2019; Yue et al., 2020). Three and two *LBDs* were up and downregulated during the AR formation, respectively. The expression patterns of *Potri.001G081400* (best hit to *AT1G31320.1*, *LBD4*), *Potri.008G072800* (best hit to *AT2G30130.1*, *LBD12*), and *Potri.007G039500* (best hit to *AT5G66870.1*, *LBD36*) were up-regulated during AR development, and were increased up to 8.5, 13.6, and 2.5 times, respectively (Figure 4). NACs, AP2, and SRSs families are also involved in many aspects of plant organ development such as root stem cell development and cell division (Li, 2021). A total of 27 *NACs* were differentially expressed during the AR development, of which 15 and 12 were up and downregulated, respectively. Five *AP2* members were differentially expressed, four of which were upregulated. Two *SRSs* exhibited upregulated expressing profiles. The results indicated that these TFs may play regulatory roles during the AR formation in poplar cuttings.

**TABLE 2:** Number of different TFs in common DEGs.

Family	Number	Up	Down
MYB	37	13	24
ERF	36	8	28
NAC	27	15	12
bHLH	27	15	12
C2H2	13	9	4
bZIP	11	10	1
HD-ZIP	10	6	4
GRAS	9	5	4
WRKY	8	5	3
Dof	7	5	2
G2-like	6	1	2
LBD	5	3	2
AP2	5	4	1
Trihelix	4	0	4
E2F/D*p*	4	2	2
GATA	4	3	1
B3	4	3	1
MYB_related	4	4	0
ARF	3	1	2
C3H	3	1	2
MIKC_MADS	3	2	1
DBB	3	3	0
SRS	2	2	0
Nin-like	2	1	1
HSF	2	2	0
TCP	2	2	0
CPP	2	2	0
FAR1	2	2	0
RAV	2	0	2
EIL	1	0	1
SBP	1	1	0
NF-YC	1	1	0
NF-YA	1	0	1
NF-YB	1	0	1
GeBP	1	1	0
TALE	1	1	0
STAT	1	1	0
BES1	1	1	0
WOX	1	1	0
GRF	1	1	0
ARR-B	1	0	1
M-type_MADS	1	0	1

*Small auxin up RNA* (*SAUR*) is the largest plant-specific family of auxin-responsive factors, which respond to early auxin signals and regulate auxin transport (Spartz et al., 2012; Li, 2021). In this study, four and three *SAUR*s were up and downregulated, respectively. *Potri.009G125900* (best hit to *AT1G75590.1*, *SAUR52*) showed an elevated expressional profile during the AR formation, and the final expression level was increased 12.7 times (Figure 4). Auxin-responsive GH3 family members regulate auxin homeostasis by synthesizing auxin conjugates in the higher plant (Li, 2021). The expression level of *Potri.003G161300* (best hit to *AT1G28130.1*, *GH3.17*) was increased 12 times at 10 d. *PIN-Form* (*PIN*s) were auxin efflux carrier family protein (Forestan and Varotto, 2012; Yang et al., 2019). We found three *PIN*s showed elevated expressional profiles. *Potri.005G187500* (best hit to *AT1G77110.1*, *PIN6*) and *Potri.002G072200* (best hit to *AT1G77110.1*, *PIN6*) were homologous genes and showed the same increasing tendency. *Potri.016G035300* (best hit to *AT1G73590.1*, *PIN1*) was up-regulated at 2 days and decreased slightly at 8 days. *ATP-binding cassette* (*ABC*) transporters positively regulate auxin efflux and influence basipetal auxin transport in the root (Titapiwatanakun et al., 2009). We found that an ABC subfamily gene *Potri.006G123900* (best hit to *AT2G36910.1*, *ABCB1*) exhibited an upregulated expression pattern during AR formation and peaked at 6 d (Figure 4). *LIKE AUXIN RESISTANT* (*LAX*) and *AUXIN RESISTANT* (*AUX*s) act as auxin transports; only *Potri.004G172800* (best hit to *AT2G21050.1*, *LAX2*) shows an upregulated expression pattern during AR formation (Swarup and Bhosale, 2019; An et al., 2020; Li, 2021). The expression level of *Potri.008G161200* (best hit of *AT4G14550.1*, *IAA14*) was increased up to 4.9, 20.9, 24.2, 50.7, and 30.4 times on different days, which was a member of the Aux/IAA protein family and involved in lateral root development (Gehlot et al., 2015; Yang et al., 2019). From 0 to 10 d, the expression of *Potri.011G042400* (best hit to *AT3G62980*, TIR1) increased continuously, which may be located upstream of the auxin regulatory pathway (Shu et al., 2019). The above results showed that these auxin signaling-related genes may involve in the formation of ARs.

The DEGs related to root development included two homolog genes of *lateral root primordium 1* (*LRP1*), one *root meristem growth factor* (*RGF9*) and one *auxin-induced in the root* (*AIR12*) (Figure 4). Three rooting-associated genes (*AUR1* and *AUR3*) were elevated during the AR formation. The expression level of *Potri.009G070800* (best hit of *AT5G12330, LRP1*) was increased 4.6, 8.6, 14.2, 16.6, and 11.6 times during the AR formation process (Figure 4). The expression level of *Potri.005G087000* (best hit of *AT5G64770.1*, *RGF9*) was increased 2.3 times at 2 d. *Potri.002G249200* (best hit of *AT3G07390.1*, *AIR9*) was decreased 23.4 times at 10 days. *Potri.018G057700* and *Potri.006G235000* (best hit of *AT4G32830.1*, *AUR1*), and *Potri.006G080500* (best hit of *AT2G45490.1, AUR3*) showed elevated expressional profiles (Figure 4). The results showed that the cell division was exuberant during the development of ARs of poplar cuttings. All the DEGs that may be related to root development and auxin signal are listed in Supplementary Table S5.

**FIGURE 5:**
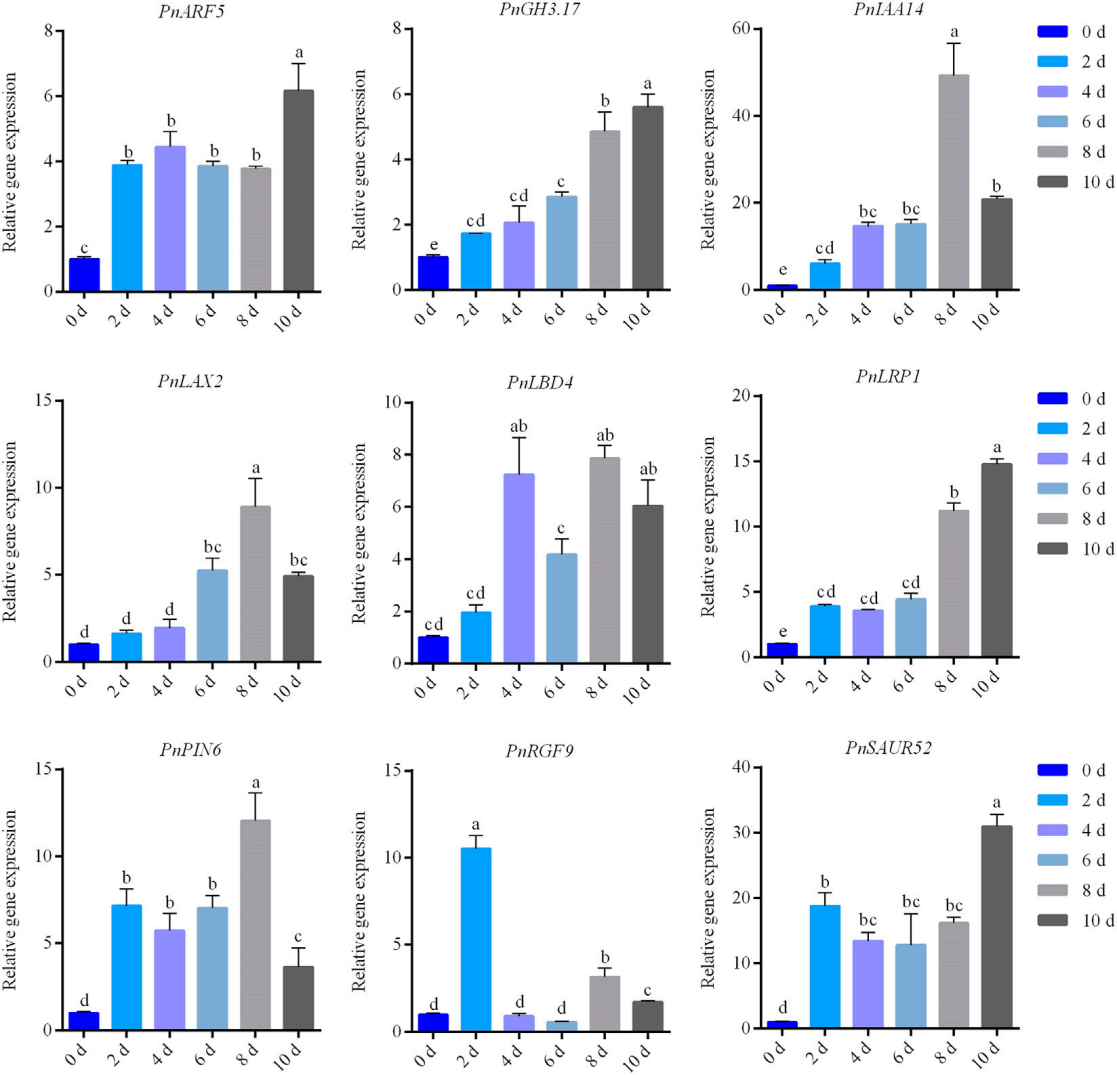
qRT-PCR results of *PnARF5*, *PnGH3.17*, *PnIAA14*, *PnLAX2*, *PnLBD4*, *PnLRP1*, *PnPIN6*, *PnRGF9*, and *PnSAUR52.*

**FIGURE 6:**
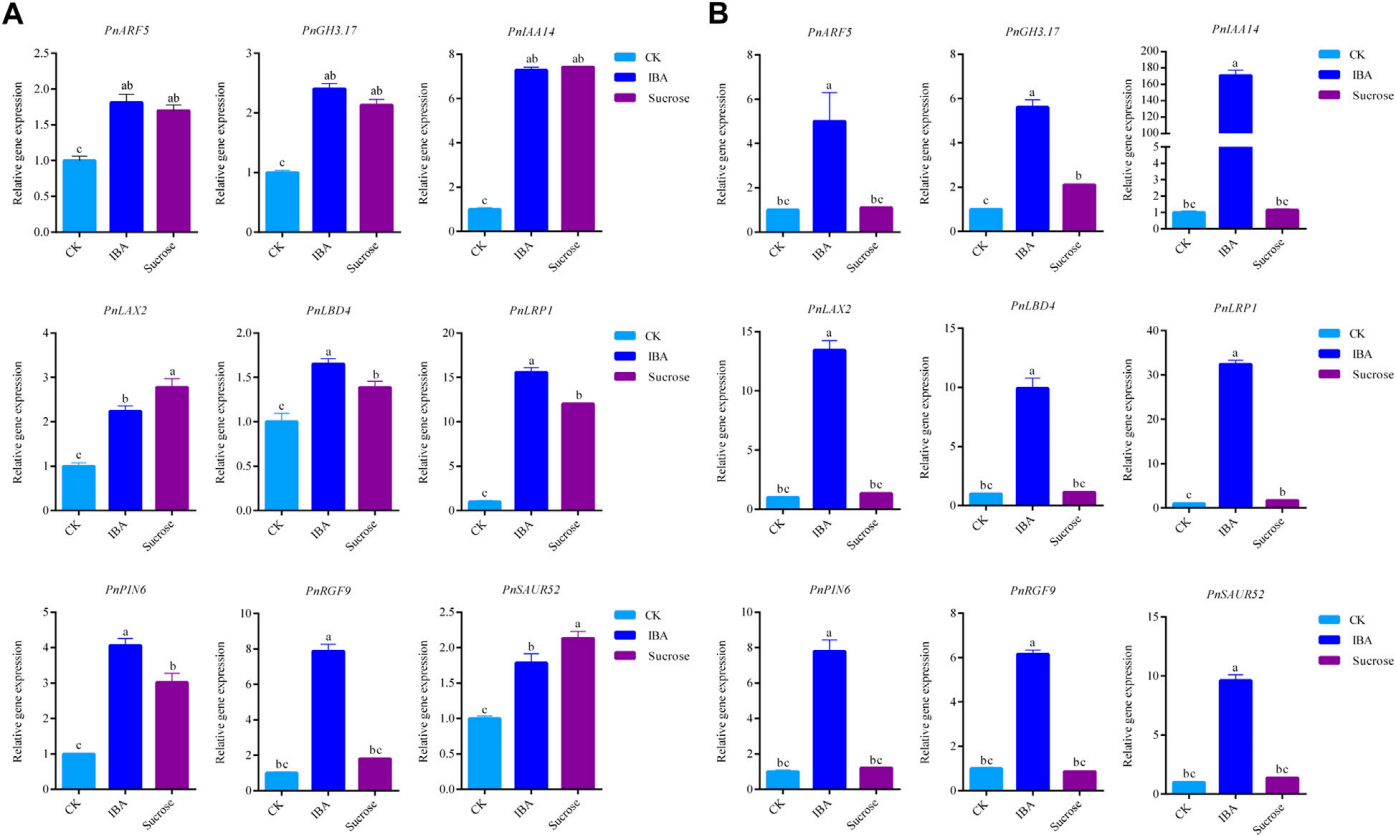
qRT-PCR results of *PnARF5*, *PnGH3.17*, *PnIAA14*, *PnLAX2*, *PnLBD4*, *PnLRP1*, *PnPIN6*, *PnRGF9*, and *PnSAUR52* under the exogenous IBA or sucrose were applied: **(A)** applied for 2 days and **(B)** applied for 4 days.

We validated the expression profiles of the genes related to rooting and auxin signals during AR formation by qRT-PCR. A total of nine candidate genes were used for qRT-PCR analysis (Figure 5), including *PnARF5*, *PnGH3.17*, *PnIAA14*, *PnLAX2*, *PnLBD4*, *PnLRP1*, *PnPIN6*, *PnRGF9*, and *PnSAUR52*. The qRT-PCR results (Supplementary Table S6) indicated that all genes showed elevated expressed profile consistent with RNA-seq. We then investigated if these genes are responsive to auxin or sucrose. We found that the expression levels of these candidate genes were significantly increased when the exogenous IBA or sucrose was applied for 2 days (Figure 6A). The expression of *PnRGF9* was increased by 3.73 times under the induction of auxin, which was significantly higher than that under the induction of sucrose (Figure 6A). The expression level of these candidate genes was significantly increased when the exogenous IBA was applied for 4 days and significantly higher than those without exogenous hormones and sucrose (Figure 6B). The expression of *PnARF5*, *PnIAA14*, and *PnLRP1* was elevated 5.92, 176.5, and 32.9 times, respectively (Figure 6B). The results implied that auxin and sucrose positively regulate AR formation by activating some common genes. But it seems that auxin plays a more effective role than sucrose in promoting AR formation in poplar. Based on the expressed profiles of these genes, we implied that all candidate genes may play critical factors in different stages of AR formation in poplar cuttings.

### Poplar cuttings with leaves exhibited improved rooting ability

**FIGURE 7:**
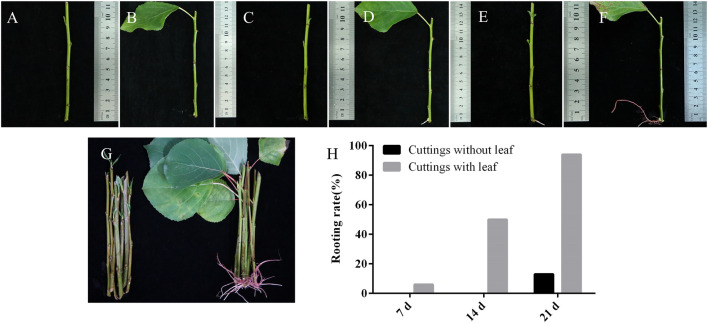
Adventitious root formation in *P. xiaohei* cuttings. **(A,C,E)** *P. xiaohei* cuttings without leaf were treated with water for 0, 14, and 21 days, respectively. **(B,D,F)** *P. xiaohei* cuttings with leaf were treated with water for 0, 14, and 21 days, respectively. **(G)** *P. xiaohei* cuttings without leaf and with leaf were treated with water for 21 days. **(H)** Rooting rates of *P. xiaohei* cuttings. Values are from 30 independent cuttings.

Photosynthesis mainly occurs in leaves and produces hormones and sugars to regulate plant growth and development (De Almeida et al., 2017). In this research, we investigated the effects of leaves on AR formation in the cuttings of poplar (Figure 7). Cuttings with and without leaves were soaked in water, respectively. For the cuttings with leaves, ARs were observed in some of them when they were soaked in the water for 7 days (Figures 7B,H). Fourteen days later, ARs with a length of around 0.5 cm were observed in 50% of them (Figures 7C,D,H). Twenty-one days later, ARs were observed in more than 90% of these cuttings (Figures 7F,H). In contrast, ARs were only observed in 13% of the cuttings without leaves when soaked in the water for 21 days (Figures 7E,H). These results indicated that cuttings with leaves significantly promoted the formation of ARs in poplar. We then shaded the leaves on the cuttings with tinfoil for 7 days and found that the rooting abilities of these cuttings were much reduced (Supplementary Figure S2). The results indicated that photosynthesis of leaves may play a role in the AR formation of poplar cuttings.

### Exogenous auxin and sucrose can promote the rooting capacity of poplar cuttings

Hormone is one of the important products of photosynthesis, in which auxin has been proven to play vital roles in regulating AR formation (Fukaki and Tasaka, 2009; Pop et al., 2011). To determine the most optimal auxin concentration for AR formation of poplar cuttings, we applied different concentrations of IBA on the top of the *P. xiaohei* cuttings and investigated the rooting abilities. The results indicated that 0.05, 0.1, 0.2, and 0.5 mg/ml IBA improved the rooting abilities to varying degrees (Supplementary Figure S3). However, 1.0 mg/ml IBA inhibited the formation of ARs. We compared the rooting rates, average AR lengths, and total AR numbers of the cuttings treated with different concentrations of IBA for 14 days, and found that 0.2 mg/ml of IBA is the optimal concentration for AR formation in *P. xiaohei* cuttings. After application of 0.2 mg/ml IBA for 14 days (Supplementary Figures S3B–D), the rooting rate can reach 80%, the average AR length reaches 3.66 ± 0.6 cm, and total AR numbers reach 4.55 ± 2.65. Carbohydrate compounds are important products of plant photosynthesis (Hyndman et al., 1981). Previous reports indicate that soluble sugar can affect the AR development of cuttings (Yu J et al., 2017). To determine the influence of soluble sugar on the AR formation of cuttings, we applied different concentrations of sucrose to the top of the cuttings without leaves (Supplementary Figure S4). The results showed that the rooting rate of cuttings reached 42%, average AR length reached 2.01 ± 2.35 cm, and total AR number reached 1.22 ± 1.92 when treated with 2.0 mg/ml (Supplementary Figures S4B–D). In contrast, the rooting rate of cuttings treated with water for 21 days was only 17% (Supplementary Figure S4D). The above results implied that applying exogenous auxin and sucrose to the top of poplar cuttings can promote rooting rates.

**FIGURE 8:**
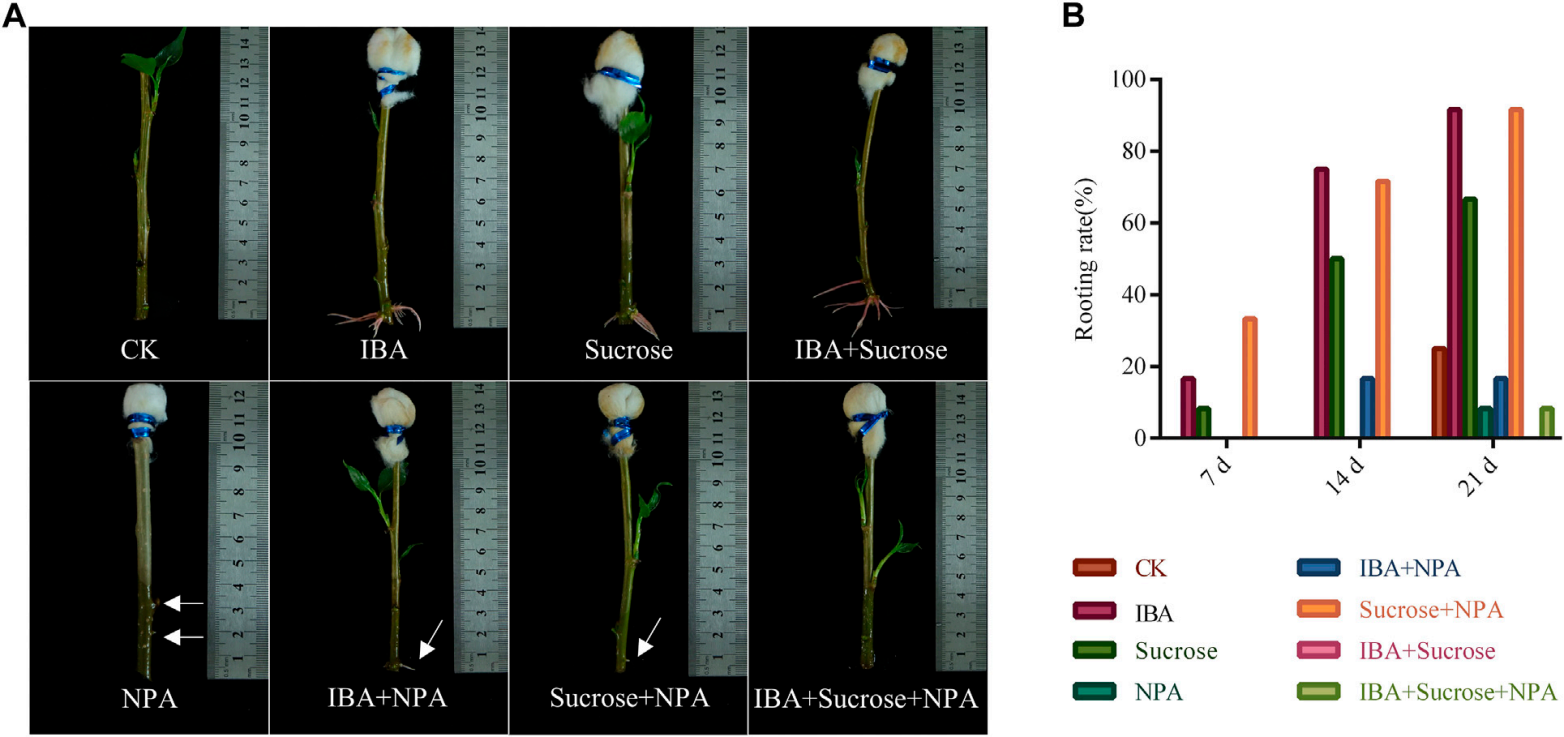
Effects of a mixture of exogenous auxin, sucrose, and NPA on root formation in *P. xiaohei* cuttings. **(A)** Mixture of exogenous auxin, sucrose, and NPA was applied on the top of *P. xiaohei* cuttings for 14 days. **(B)** Rooting rate of *P. xiaohei* cuttings treated with a mixture of exogenous auxin, sucrose, and NPA for 7, 14, and 21 d, respectively. Values are from 12 independent cuttings. White arrows represent irregular bumps and unhealthy roots.

In order to test which combination of hormones is suitable for the formation of ARs in poplar cuttings, a different mixture of IBA, sucrose, and NPA (auxin inhibitor) was applied to the top of the cuttings without leaves (Figure 8A). We applied 0.2 mg/ml IBA and the mixture of 0.2 mg/ml IBA and 2 mg/ml sucrose on the top of cuttings without leaves for 14 days and found that the rooting rates reached 75% and 71.6% (Figures 8A,B). When the mixture solution was applied for 21 days, the rooting rates of cuttings can reach 91.6% (Figure 8B). In contrast, the rooting effect of cuttings with 0.2 mg/ml NPA or NPA mixtures (0.2 mg/ml NPA + 0.2 mg/ml IBA, 0.2 mg/ml NPA + 2 mg/ml sucrose, and 0.2 mg/ml NPA + 0.2 mg/ml IBA + 2 mg/ml sucrose) applied to the top was not satisfactory (Figures 8A,B). We found that NPA can reduce the effect of auxin on promoting AR formation. Irregular bumps and unhealthy roots were observed at the base of cuttings under the treatment of 0.2 mg/ml NPA or NPA mixtures (Figure 8A). The above results implied that the difference in rooting ability between cuttings with leaves and without leaves is mainly due to the synergistic effect of auxin and organic compounds such as sugars synthesized by the leaves. When auxin synthesis or transport is inhibited, poplar cuttings have almost no AR formation or produce a small number of unhealthy ARs.

## Discussion

### Photosynthesis in leaves produces auxin and sucrose to promote adventitious root development

The main propagation method of woody plants is cuttage, which not only improves efficiency but also preserves varieties of precious traits in time (Bannoud and Bellini, 2021; Xu et al., 2021). In the actual production process, some agronomic measures such as cutting quality, cut characteristics, and cuttings with or without leaf can affect the rooting of cuttings (Hilo et al., 2017). Here we found that the difference in rooting ability between cuttings without and with leaves is mainly due to the difference in auxin levels. In addition to photosynthesis-producing hormones and nutrients to promote the formation of ARs, auxin stored in chloroplasts and cuttings’ own nutrients may also involve in the process of AR formation (Yuan et al., 2019). We discovered that the hormones and nutrients stored by the plant itself are not the main factors affecting the rooting of cuttings by shading experiment. Sucrose and starch are important products of photosynthesis, which play a critical role in the growth of different plant organs (Rapaka et al., 2005; Yuan et al., 2019). The synergistic effect of sugar and hormones on root formation has been extensively studied in recent years. For example, 2 mg/L IBA combination with adequate 20 g/L sucrose can produce roots and increase their *ex vitro* survival (Estrella-Maldonado et al., 2022). The expression levels of *CpARF*s and *CpIAA*s were elevated with increasing IBA and sucrose concentrations in the culture medium. *In vitro* papaya seedlings can also produce ARs under strong light even without the addition of auxin and sucrose, and found the same gene expression pattern in the stem base as in the leaves (Estrella-Maldonado et al., 2022). Sucrose is hydrolyzed to generate energy (Mishra et al., 2009). Sucrose can upregulate the expression of auxin-responsive factor ARFs and transporter PINs (Rolland et al., 2006; Mishra et al., 2009). At present, the molecular mechanism of the combined effect of auxin and sucrose during AR formation still needs further research.

### Polar auxin transport promoted adventitious root formation and inhibited lateral bud germination

Auxin is transported to the bottom of the cutting stem by polar transport to promote the development of ARs (Lin and Sauter, 2019). We found a phenomenon that cuttings without leaves have less ability to produce ARs but the axillary buds of which sprouted into young leaves earlier than cuttings with leaves. Different concentrations of IBA applied to the tips of cuttings without leaves can not only promote the formation of ARs but also inhibit the germination of axillary buds. Leaves produced more auxin through photosynthesis, and the auxin was polarly transported to the base of the cuttings. We proposed a hypothesis that auxin molecules enter the ungerminated axillary bud cells by means of carrier cooperative transport or free diffusion, and undergo *de novo* synthesis in axillary bud cells. It was found that the contents of IBA and L-tryptophan in axillary buds of cuttings with leaves were significantly higher than those of cuttings without leaves (Supplementary Table S7). In addition, the levels of inactive covalent compounds of auxin were significantly increased, including IAA-Leu, IAA-Glu, IAA-Asp, and IAA-Ala. Only a small amount of auxin exists in free-state IAA in plants, and most of them exist in the form of non-covalent compounds. These inactive covalent compounds can be converted to IAA and are important products of auxin metabolism. When a large amount of IAA or inactive covalent compounds of IAA is transported to the base of the cuttings, the auxin is synthesized *de novo* and induced ARs. The contents of IBA at the base of cuttings were significantly less than those of axillary buds, which may be due to the conversion of IBA to IAA. The contents of L-tryptophan in the base showed the same contrast with the axillary buds. The contents of L-tryptophan in cuttings with leaves were nearly three times that in cuttings without leaves. L-tryptophan is currently known as an important precursor substance in the three auxin synthesis pathways, and its level will affect the contents of auxin synthesis (Hagen, 2015). We detected two substances, IPA and IAN, in the base of the cuttings with leaf. These two substances are the intermediate products of the Trp-IPA-IAAld-IAA and Trp-IAOx-IAN-IAA pathways, respectively. However, these two substances were not detected in the base of the cuttings without leaves. These results indicated that the significant difference in auxin levels may be the main reason for the AR formation in poplar cuttings.

### Potential mechanisms involved in the AR formation process

Several genes involved in AR formation were obtained in our transcriptome results, and these genes showed an elevated expression pattern after the application of IBA and sucrose. Some of these genes which are mediated by auxin to affect root formation and development have been reported. For example, ARF5/MONOPTEROS (MP) positively regulates the expression of *miR390* in the root meristem through binding auxin response elements (AuxREs) located in the *miR390* promoter (Ghosh et al., 2019). In our results, *PtARF5* was upregulated throughout the AR formation process of polar. LRP1 was reported to participate in the development of lateral root primordia by mediating the expression of downstream *YUC4* in *Arabidopsis* (Singh et al., 2020). We found that *PtLRP1* also increased significantly in poplar cuttings after 8 d treatment. These results indicated that ARF5 and LRP1 may play a conserved role during AR formation. The expression level of *RGF9* was significantly increased at early stages of AR development after applying IBA in highbush blueberry (*Vaccinium corymbosum* L.) (An et al., 2020). In our RNA-seq results, we found the expression level of *PtRGF9* was increased 2.3 times at 2 d and then started to decrease. Yordanov et al. (2010) reported that LBD1 can regulate secondary xylem development and was regulated by auxin during xylem development in *P. tremula* × *P. alba*. The expression level of *PtLBD1*was elevated during AR formation in our results. The result indicated that *LBD* may play a role in AR formation by regulating lignin biosynthesis. Yu M et al. (2017) revealed that *PIN1*, *ABCB1*, and *AUX2* involved in auxin transport were highly expressed in hybrid poplar treated with exogenous IAA. We found similar expression patterns for *PIN1*, *ABCB1*, and *AUX2* in our results. These results indicated that the remaining leaves on the cuttings may trigger some biological processes involved in AR formation. We found that *Potri.004G111400* (*PtAAAP19*) which has been identified as a candidate gene by the QTL mapping method was up-regulated during AR formation in our results (Sun et al., 2019).

### Genes related to light signal co-expressed with TF during AR formation

In RNA-seq results, we identified some DEGs associated with rooting and auxin signals. Of these genes, ARFs have been reported to mediate the auxin signaling pathway, thereby affecting plant AR formation and development (Liu et al., 2020; Li, 2021). ARF5 showed an elevated expressional profile during AR formation. *Potri.001G027700*, *Potri.002G092700*, *Potri.001G288301*, *Potri.001G011300*, and *Potri.019G057500* highly expressed in root cell and related to AR initiation and elongation. Auxin signal transduction has the function of maintaining hormone homeostasis during AR development (Guan et al., 2019). Potri.016G035300 belongs to the PIN family and encodes an auxin efflux carrier involved root development (Yang et al., 2019). Potri.006G123900 mediates cellular efflux of IAA and interacts with PIN genes that may influence auxin transport in the root (Jenness et al., 2022). Potri.008G098850 plays a negative regulator of auxin polar transport inhibitors and regulates auxin distribution and homeostasis in roots (Jenness et al., 2022). Some organic synthases were elevated during AR development. *Potri.003G176900*, *Potri.014G145100*, *Potri.001G051500*, *Potri.001G051600*, and *Potri.003G176700* encode chalcone synthase (CHS), a key enzyme involved in the biosynthesis of flavonoids (Li and Zachgo, 2013). Flavonoids are involved in the regulation of auxin transport and the modulation of root gravitropism (Li and Zachgo, 2013). The developmental pattern of ARs is similar to that of lateral roots (LR) (Singh et al., 2020). *Potri.008G161200* and *Potri.009G070800* mediate primary root meristem initiation and involved LR development (An et al., 2020; Singh et al., 2020). Among these genes related to root and auxin signal, we found two NACs co-expressed with ARF5, *Potri.005G098200* and Potri.007G065400, encode a transcription factor involved in the shoot apical meristem formation and mediated LR formation (Chen et al., 2016). Photosynthesis can affect the accumulation of photosynthetic products and the growth of organs in plants (Rapaka et al., 2005). The BP and KEGG pathway indicated that genes related to photosynthesis may be involved in the formation of ARs in leafy cuttings from poplar. *Potri.011G126700* and *Potri.001G407100* are the main components of light-harvesting chlorophyll a/b–protein complex and are involved in the process of chlorophyll absorbing light (Zubo et al., 2018). The expression of *Potri.011G126700* and *Potri.001G407100* increased from 18.15 to 149, 21.67 to 107.69, respectively. Photosystem I light-harvesting complex gene 6 (LHCA6) responds to red and blue lights and co-expressed with BBX32 (Zubo et al., 2018). *Potri.009G140500* is involved in the chloroplast signaling recognition particle pathway targeted by LHCP and response to bright light (Holtan et al., 2011). We implied the above genes may play critical roles in the molecular mechanism of ARs development in *P. xiaohei* cuttings.

## Data Availability

The datasets presented in this study can be found in online repositories. The names of the repository/repositories and accession number(s) can be found in the article/Supplementary Material.

## Abbreviations

ACC, 1-aminocyclopropanecarboxylic acid; AR, adventitious root; d, day; egg NOG, evolutionary genealogy of genes; GO, gene ontology; IAA, indole-3-acetic acid; IAA-Ala, N-(3-indolylacetyl)-L-alanine; IAA-Glu, indole-3-acetyl glutamic acid; IAA-Leu, N-(3-indolylacetyl)-L-leucine; IAN, 3-indoleacetonitrile; IBA, indole-3-butyric acid; IPA, 3-indolepropionic acid; KEGG, Kyoto Encyclopedia of Genes and Genomes; LC-MS, liquid chromatography–mass spectrometry; LR, lateral root; oxIAA, 2-oxindole-3-acetic acid; TRP, L-tryptophan.
