# Supplementary figures and images for: Transcriptomic profiles of poplar (Populus simonii × P. nigra) cuttings during adventitious root formation
Source: Front Genet. 2022 Sep 8;13:968544. doi: 10.3389/fgene.2022.968544 (PMC9493132; doi:10.3389/fgene.2022.968544)

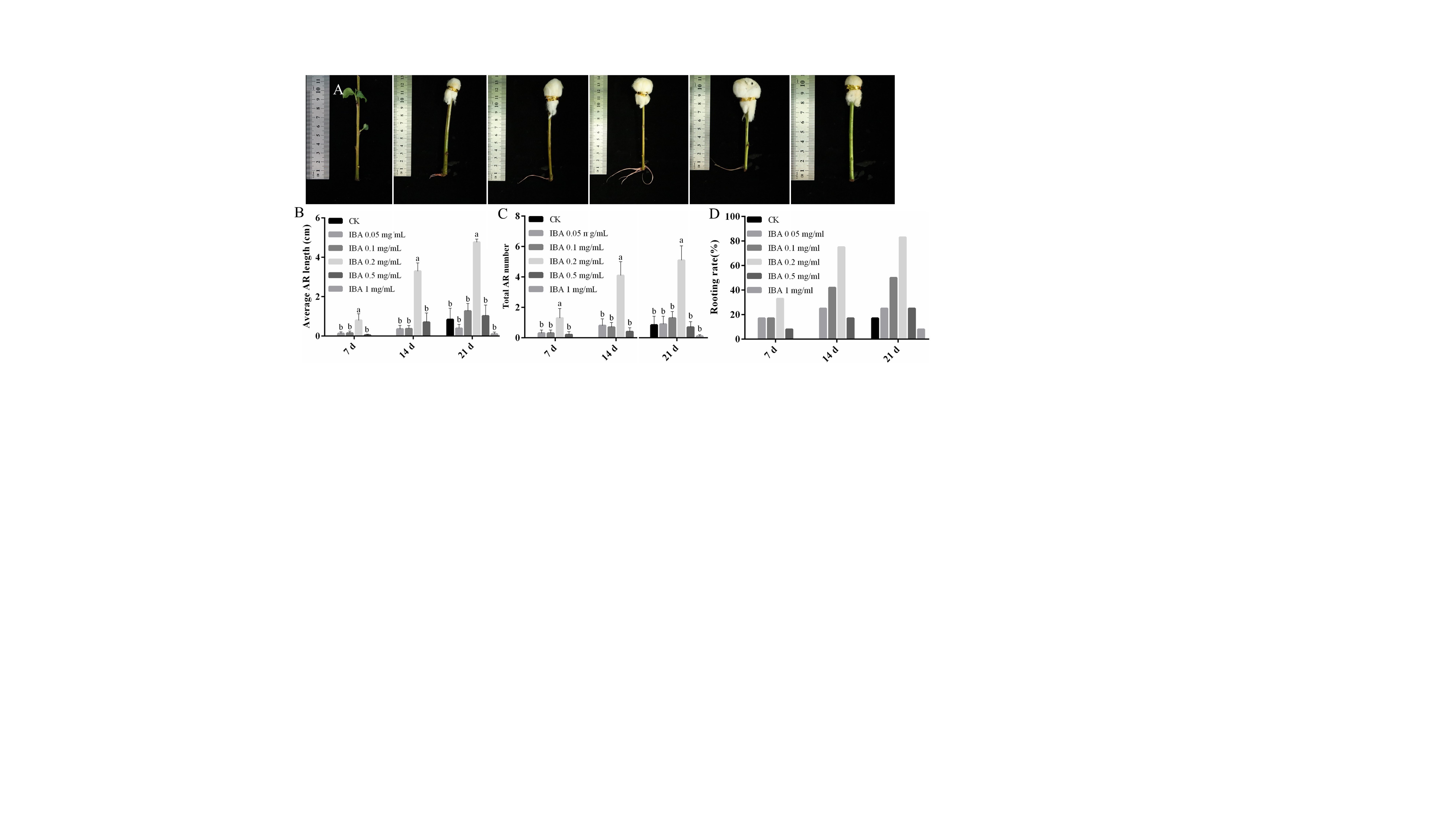

Supplement: Supplementary file 1 [file Image3.JPEG]

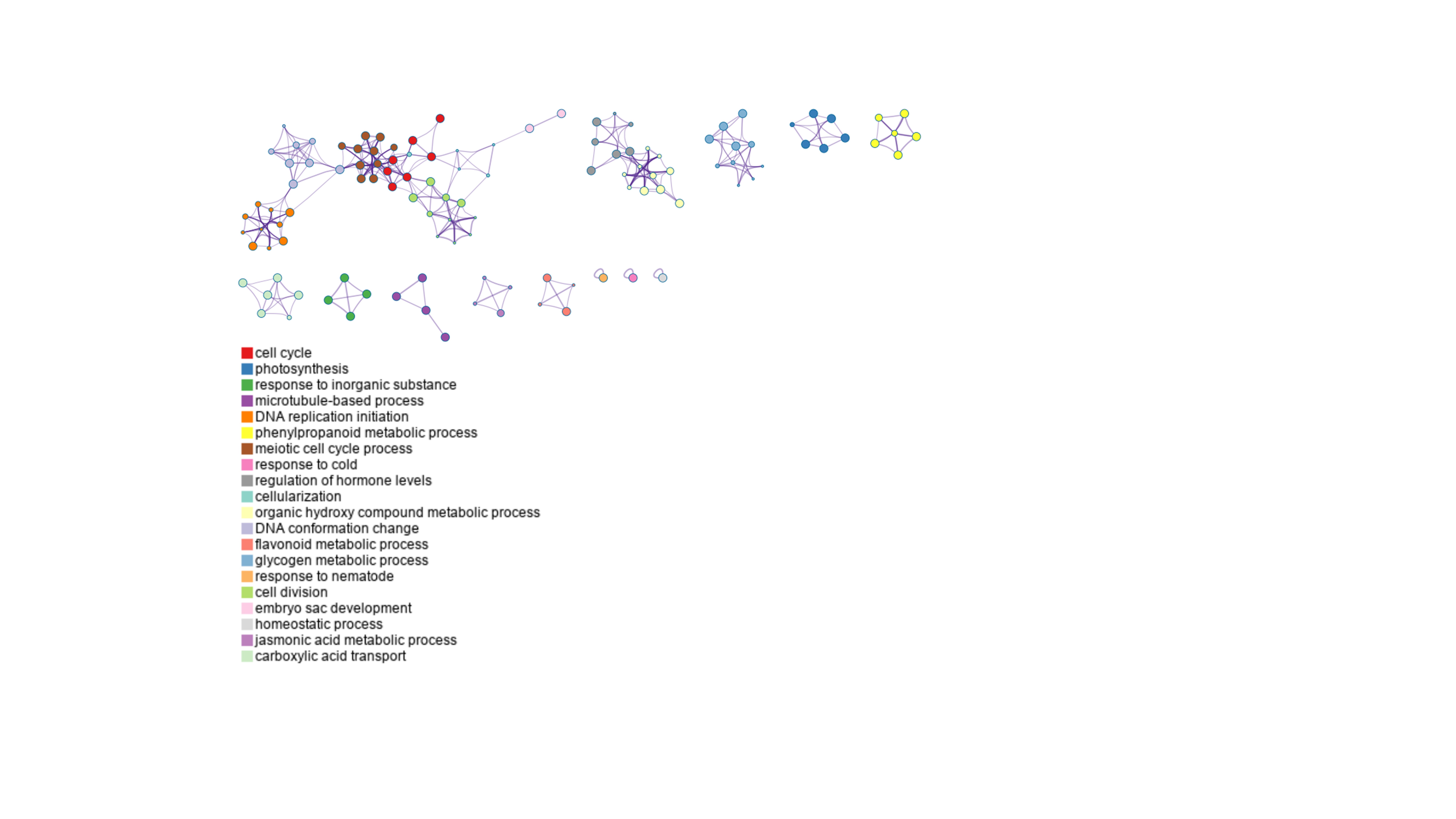

Supplement: Supplementary file 3 [file Image1.JPEG]

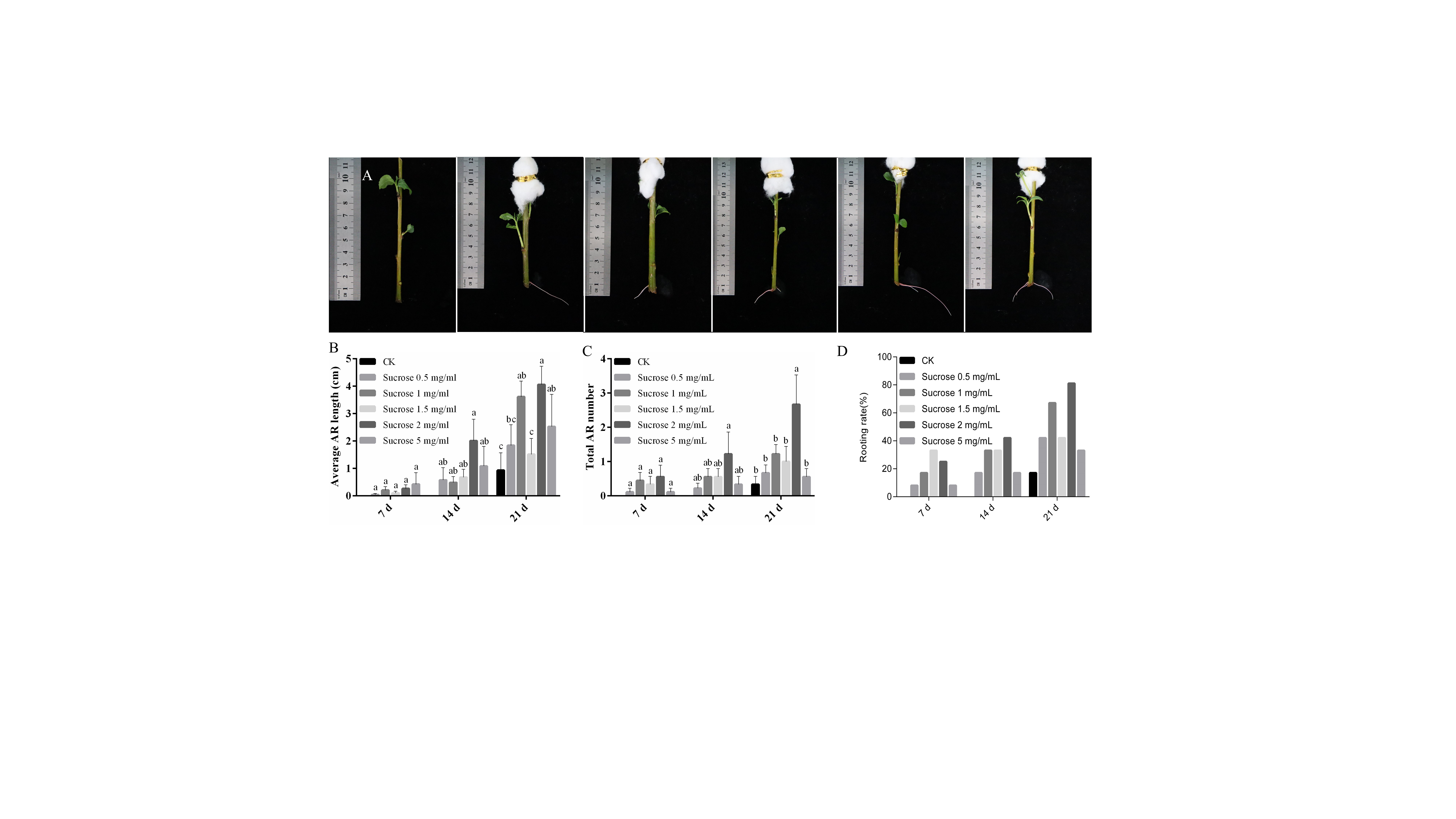

Supplement: Supplementary file 4 [file Image4.JPEG]

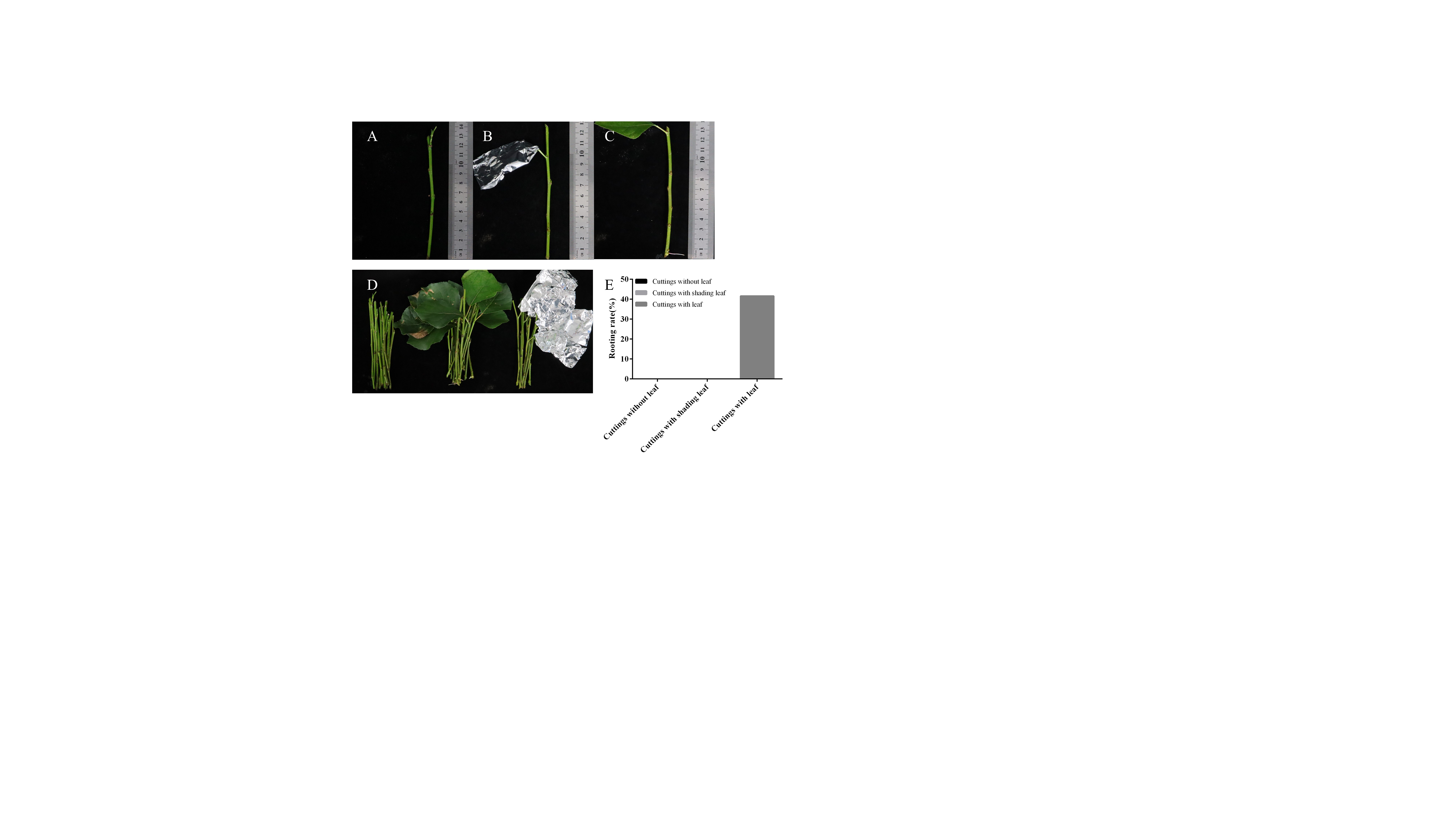

Supplement: Supplementary file 5 [file Image2.JPEG]
