# Supplementary material for: Transcriptomic profiles of poplar (Populus simonii × P. nigra) cuttings during adventitious root formation
Source: Front Genet. 2022 Sep 8;13:968544. doi: 10.3389/fgene.2022.968544 (PMC9493132; doi:10.3389/fgene.2022.968544)
Supplement: Supplementary file 2 [file Table8.DOCX]

**Table S8 Primers used for the qRT-PCR analysis.**

| Gene name | Gene ID | 5’-Primer sequence | 3’ -Primer sequence |
| --- | --- | --- | --- |
| ARF5 | *Potri.002G024700* | TTCAAGACCAGTCTGGGACAC | GGCACAGTAACTGAGAAGGGA |
| GH3.17 | *Potri.003G161300* | CAATGCATGTCAAATACAGCGA | AGCTTGTGAGGAGCTCAGTTA |
| IAA14 | *Potri.008G161200* | ATGGTGCACCCTATCTTCGC | GGCGCTTGCATGAATTGACA |
| LAX2 | *Potri.004G172800* | TGTGGGTGCTGATTGTTGGG | CAAGGGGTGCGAGTATGGTTT |
| LBD4 | *Potri.001G081400* | CCCAAGATTGCGTGTTTGCC | CCATACACTGGATCACGCAC |
| LRP1 | *Potri.009G070800* | AGTGACGGCAGTGGAAGATG | CCGTTTCTCCCTACGCCATT |
| PIN6 | *Potri.005G187500* | GCAAAGATGTGCCAGGCAAG | CCAGAGAAGCCCCAAGACAC |
| RGF9 | *Potri.005G087000* | GCAAGGACTTTGCGAGAGGT | TGGAGGCTTCTTACTTGCTGG |
| SAUR52 | *Potri.009G125900* | TCTGTGTAGGAGCCAGTTGC | TCGACACGACCCGGATGAT |
