# Supplementary material for: Transcriptomic profiles of poplar (Populus simonii × P. nigra) cuttings during adventitious root formation
Source: Front Genet. 2022 Sep 8;13:968544. doi: 10.3389/fgene.2022.968544 (PMC9493132; doi:10.3389/fgene.2022.968544)
Supplement: Supplementary file 7 [file Table2.DOCX]

**Table S2 GO enrichment was performed on these common DEGs to identify biological processes (BP) involved in the AR formation.**

| GO biological process complete | Number | Fold enrichment |
| --- | --- | --- |
| cell cycle  root development | 119  76 | 2.47  1.82 |
| auxin transport | 23 | 3.12 |
| cellular hormone metabolic process | 17 | 3.91 |
| metabolic process | 1395 | 1.23 |
| photosynthesis | 52 | 2.56 |
| response to inorganic substance | 140 | 1.8 |
| microtubule-based process | 56 | 2.37 |
| regulation of hormone levels | 60 | 2.13 |
| flavonoid metabolic process | 43 | 2.96 |
| photosynthesis, light, reaction | 37 | 2.53 |
| organic acid transport | 28 | 3.34 |
| cell division | 44 | 2.67 |
| jasmonic acid metabolic process | 17 | 4.46 |
