# Supplementary material for: Transcriptomic profiles of poplar (Populus simonii × P. nigra) cuttings during adventitious root formation
Source: Front Genet. 2022 Sep 8;13:968544. doi: 10.3389/fgene.2022.968544 (PMC9493132; doi:10.3389/fgene.2022.968544)
Supplement: Supplementary file 10 [file Table6.DOCX]

**Table S6 Genes related to rooting and auxin signal during AR formation detected by qRT-PCR**

| Gene | 0d | 2d | 4d | 6d | 8d | 10d |
| --- | --- | --- | --- | --- | --- | --- |
| *PnARF5* | 1.00±0.15^c^ | 3.89±0.15^b^ | 4.45±0.46^b^ | 3.86±0.15^b^ | 3.78±0.08^b^ | 6.17±0.84^a^ |
| *PnGH3.17* | 1.00±0.07^e^ | 1.73±0.01^cd^ | 2.06±0.52^cd^ | 2.86±0.14^c^ | 4.86±0.60^b^ | 5.60±0.40^a^ |
| *PnIAA14* | 0.99±0.07^e^ | 6.07±0.84^cd^ | 14.67±0.88^bc^ | 15.10±1.15^bc^ | 49.25±7.43^a^ | 20.87±0.59^b^ |
| *PnLAX2* | 1.00±0.04^d^ | 1.63±0.19^d^ | 1.96±0.49^d^ | 5.26±0.71^bc^ | 8.91±1.60^a^ | 4.93±0.22^bc^ |
| *PnLBD4* | 1.00±0.06^cd^ | 1.96±0.30^cd^ | 7.25±1.40^ab^ | 4.18±0.59^c^ | 7.86±0.50^ab^ | 6.05±1.00^ab^ |
| *PnLRP1* | 1.00±0.06^c^ | 3.89±0.15^cd^ | 3.58±0.08^cd^ | 4.45±0.46^cd^ | 11.19±0.61^b^ | 14.76±0.42^a^ |
| *PnPIN6* | 1.00±0.07^d^ | 7.15±1.00^b^ | 5.75±0.97^b^ | 7.03±0.73^b^ | 12.05±1.61^a^ | 3.64±1.12^c^ |
| *PnRGF9* | 0.99±0.08^d^ | 10.51±0.76^a^ | 0.90±0.15^d^ | 0.56±0.06^d^ | 3.16±0.49^b^ | 1.72±0.08^c^ |
| *PnSAUR52* | 0.99±0.08^d^ | 18.77±2.04^b^ | 13.40±1.31^bc^ | 12.80±4.79^bc^ | 16.20±0.83^bc^ | 30.94±1.79^a^ |

The 2^−ΔΔCT^ method was used to analyze the expression levels of *PnXXXs*. The values of log_2_ (sample/control) upon to different treatment conditions were calculated as the relative expression levels. The statistic significant differences of the expression levels were analyzed using Duncan's multiple range test method (*P* <0.05). All the values were compared with the corresponding values. Results are the mean ± SD of three replicates, and “abc” represents statistic significant differences (*p*< 0.05).
